# A Manual of Therapeutics

**Published:** 1895-03

**Authors:** 


*A Manual of Therapeutics.* By A. A. STEVENS, A.M., M.D. Pp. 435. Philadelphia: W. B. SAUNDERS. 1894.

This addition to the numerous manuals of therapeutics is distinctly a good one. It does not aim at doing more than serve to the student as an introduction to the large text-books. Without going so far as to say that it is superior to those already in use, it is to be recommended as being systematic, clear, concise, very fairly up to date, and carefully indexed. The division on applied therapeutics will be useful to junior practitioners as well as to students. A second edition will give an opportunity of removing some few slight faults and infelicities of expression.

